# An organizing framework to break down Western-centric views of knowledge in North–South research

**DOI:** 10.1007/s11625-024-01478-6

**Published:** 2024-02-17

**Authors:** Hannah Turner, Briony Rogers, Sarah Kneebone, Diego Ramirez, Matthew French, Mere Jane Sawailau, Filise Volavola, Sholyn Baran, Kelera Matavesi, Orlando Newton, Maraia Batiota Luveniyali, Autiko Tela, Isoa Vakarewa

**Affiliations:** 1 Monash Sustainable Development Institute, Monash University, Melbourne, Australia; 2 Faculty of Art, Design and Architecture, Monash University, Melbourne, Australia; 3 Revitalising Informal Settlements and Their Environments (RISE), Suva, Fiji; 4 Live and Learn, Suva, Fiji; 5 University of the South Pacific, Suva, Fiji; 6 Fiji National University, Suva, Fiji

**Keywords:** Interdisciplinary, North–South, Knowledge co-construction, Diverse ways of knowing, Research framework

## Abstract

Global challenges, such as climate change, persistent poverty, and food insecurity are complex problems. These societal, environmental, and economic challenges cross scientific disciplines, communities, and geographies, requiring interdisciplinary, North–South solutions. Nevertheless, prevailing sustainability science responses are Western-centric. Some seminal studies have attempted to understand and engage with diverse knowledge systems. These include decolonial and Indigenous methodologies, such as “Two-Eyed Seeing”, which emphasizes the importance of using both Western and Indigenous knowledge to gain a more comprehensive understanding of the world, and participatory action research, which highlights the importance of involving participants in the research process and promoting social change through collaborative effort. However, apart from in-country research collaborations with traditional Indigenous knowledge, most North–South studies overlook the role or influence of Western-centric views and therefore fail to recognize and incorporate diverse worldviews and knowledge systems. This may, in part, reflect the tendency to categorize research into disciplinary silos, but more likely is the unintentional, yet prevalent, view that Western science is “objective and neutral.” As more scholars from multiple disciplines and geographies focus on interdisciplinary North–South research, it is critical that researchers reflect on dominant research approaches and knowledge production. Studies can co-construct, reproduce, or control the forms of knowledge generated—whether intentional or unintentional. This paper presents an organizing framework to help researchers navigate, understand, and engage with diverse forms of knowledge in undertaking North–South research. The framework draws on empirical observations from the authors’ interdisciplinary research and from empirical cross-cultural literature. It comprises three contextual levels of influence, featuring guiding principles and subsequent practical actions researchers can use to navigate the complexities of knowledge co-construction in North–South research.

**Supplementary Information:**

The online version contains supplementary material available at 10.1007/s11625-024-01478-6.

## Introduction

Global challenges, such as climate change, lack a single unified solution and also cannot be resolved from one viewpoint or approach (Kapucu and Beaudet 2020; Lodge and Wegrich 2014; Walls 2018). These societal, environmental, and economic challenges cross scientific disciplines, communities, and countries; thus, interdisciplinary North–South solutions are required to tackle these seemingly impossible tasks (Australian Public Service Commission 2018; Commission for Research Partnerships with Developing Countries [KFPE] 2018; Dentoni et al. 2018; Kotze and Dymitrow 2021; Lodge and Wegrich 2014).

Despite challenges associated with the term North–South, such as its oversimplification of the developmental, social and economic histories (Dados and Connell 2012), geopolitical boundaries (Horner 2020), and its inaccuracy in relation to Australia and New Zealand’s location in the Southern Hemisphere, it endures in categorizing low- and middle-income countries (primarily situated geographically south) and industrialized and higher-income countries (north of the equator). While these terms perpetuate Western and Eurocentric epistemologies (Connell 2007, 2014; Kothari and Cooke 2001), their inclusion in this paper intends to leverage existing terminology for enhanced searchability. However, in alignment with this article's objective, the term South–North will be used throughout the body of the article to better align with its aim.

Although diverse epistemologies (ways of knowing) are the reality of interdisciplinary research, most South–North research has been conducted on “others”—by and for Western benefit—under the assumption that there is a knowable reality to be extracted (Gone 2018; Kotze and Dymitrow 2021; Schmidt and Pröpper 2017; Windchief et al. 2017). Euro-American-centric perspectives have led to the dominance of Western knowledge and resulted in the misrepresentation of other cultural ways of knowing (Brandt et al. 2013). The perceived universality of Western knowledge limits the exploration, collaboration, and recognition of diverse viewpoints—the cornerstone of interdisciplinary research (Anthony-Stevens and Matsaw 2019; Brandt et al. 2013; Hopkins et al. 2019; KFPE 2018; Kotze and Dymitrow 2021; Lodge and Wegrich 2014; O’Flaherty et al. 2008; R’boul 2020; Sastry and Ramasubramanian 2020; Schmidt and Pröpper 2017; Smith 2012; Windchief et al. 2017). Ways of knowing encompasses diverse methods and avenues through which individuals acquire knowledge and understand the world, including work, experience, and time (Harris 2007). Various cultures and academic disciplines may recognize and prioritize distinct ways of knowing.

Within the dynamic landscape of academic inquiry, there is a crucial need for more interdisciplinary research. As scholars from diverse fields engage in North–South interdisciplinary research, synthesizing the knowledge co-construction is essential to promote the adoption of decolonial and Indigenous research practices. Seminal contributions have been made on the ethical implications of the hegemonic and colonial nature of research (Anthony-Stevens 2020; Beckford 2018; Cameron et al. 2021; Castleden et al. 2017; Lipscombe et al. 2021; Matson et al. 2021; McNamara and Naepi 2018), decolonial research ethics (Decoloniality Europe 2013; Matias 2021), Indigenous and decolonizing studies (Smith et al. 2018; Wooltorton 2023), and feminist studies (Arashiro and Barahona 2015; Manning 2018).

While scholars have explored the dynamics of South–North research collaboration, few frameworks have gone beyond research cooperation to operationalize South–North knowledge co-construction (Anthony-Stevens and Matsaw 2019; Broesch et al. 2020; KFPE 2018; Leonard and Mercier 2016; Sylvester et al. 2020). Extant literature emphasizes the values and principles of South–North collaboration, namely, the need for empowerment, joint decision-making, and equitable power and resource distribution (Dentoni et al. 2018; Jentsch 2004; Waddell et al. 2015). However, effective knowledge co-construction involves more than equal opportunity, it requires deconstruction of researcher positionality (Anthony-Stevens and Matsaw 2019; Bozhkov et al. 2020), and dismantling the conscious and subconscious ways in which knowledge is generated (Adams et al. 2017; LaVallie and Sasakamoose 2021; Lipscombe et al. 2021; Zurba et al. 2019). Insufficient articulation of *how* to deconstruct and incorporate diverse ways of knowing perpetuates Western knowledge constructs (Kotze and Dymitrow 2021; Schmidt and Pröpper 2017), hindering effective collaboration with knowledge partners and representation of diverse knowledge systems.

An illustrative example of what can happen when there is a failure to ‘appreciate the merits of the traditional knowledge’ is the Asian Development Bank and the Indonesian Ministry of Agriculture efforts to boost rice production in Indonesia in 1970 (Lansing and Fox 2011, p. 931). In this case study, colonial advisors to the Asian Development Bank recommended incentivizing farmers to cultivate high-yield rice varieties, which produced more grain than the native Balinese rice and enabled farmers to plant more frequently. However, their policy recommendations failed to recognize the Balinese religious water temples, tika calendar, and cooperative practices employed by farmers in pest and water management. This oversight resulted in water shortages and an exacerbated pest problem—a core factor in the 1982–1985 decline in Balinese rice yields (Lansing 2009; Lansing and Fox 2011).

The complexity of global problems necessitates intentional efforts to decolonize research and incorporate diverse ways of knowing (Jentsch 2004; Kotze and Dymitrow 2021). Knowledge production is context dependent, i.e., it depends on individual, social, and environmental contexts (Anthony-Stevens and Matsaw 2019; Hall and Callery 2001; Spretnak 2011). It is developed through the accumulation of collective experiences, discursive assumptions, and meaning making (Pham and Gothberg 2020; LaVallie and Sasakamoose 2021). Knowing can be constructed, decoded, and experienced in numerous ways (Chambers 2007; Ebersöhn and Malan-Van Rooyen 2018; Hart et al. 2017; Mazzetti and Blenkinsopp 2012) and is shaped through social and institutional norms and structures (Pham and Gothberg 2020; LaVallie and Sasakamoose 2021). This diversity brings nuance in viewpoints and ways of knowing, potentially unfamiliar to researchers new to South–North research (Hopkins et al. 2019; O’Flaherty et al. 2008; Smith 2012; Windchief et al. 2017). Moreover, the lack of research frameworks to assist researchers working across geographies limits the generation of South–North knowledge co-construction (Broesch et al. 2020).

As global challenges continue to escalate, and more scholars from multiple disciplines focus on South–North research, it is essential for Western researchers to grasp the intricacies of colonialism and Western-centric knowledge, and what it means to explore and engage with diverse viewpoints and knowledge systems (Jentsch 2004).

Given the increasing pressure to solve these pressing global problems, in this paper, an organizing framework is proposed to investigate and identify practical actions researchers can take to engage with diverse forms of knowledge. The goal of this paper is not to review or criticize past literature on colonial research, but to provide a series of recommendations to help interdisciplinary researchers new to South–North research navigate the process of exploring and integrating unique and contextual ways of knowing in order to ensure they represent diverse and conflicting viewpoints in such research (Anthony-Stevens and Matsaw 2019; Broesch et al. 2020). As authors we recognize our privilege as researchers, many with backgrounds informed by colonial histories, yet hope that this framework can offer the foundations for increased adoption of knowledge co-construction in South–North research.

## Materials and methods

### Research design

This research comprises empirical participatory-observational research and a systematic configurative review. Participatory observations in the lead author’s empirical research revealed a gap in their knowledge surrounding South–North research collaboration, leading to a review of literature to seek and develop guidance, following PRISMA principles (Page et al. 2021). The review employed a configurative systematic approach, coding ‘descriptive themes’ and generating ‘analytical themes’ which formulate new interpretations from the qualitative data (Thomas and Harden 2008), as used by Amicarelli and Bux (2021), Roodhuyzen et al. (2017), Skeen et al. (2022). The resulting framework aims to break down Western-centric views of knowledge in South–North research.

#### Empirical participatory-observational research

The empirical research analysis in this paper is part of a flood management study within a broader research program called Revitalizing Informal Settlements and their Environment (2021) with communities living in informal settlements in Fiji and Indonesia. Human-caused inequalities, such as urbanization, the lack of affordable housing, and the limited supply of critical infrastructure, mean most informal settlements in Fiji are built on flood-prone sites (Connell 2011). This study examined residents’ experience of flooding and their flood protection measures. Existing literature on floods suggests self-efficacy plays an essential role in protective motivation (Bandura 1977; Lewis 2010; Odidi et al. 2020; Seebauer and Babcicky 2020; van Valkengoed and Steg 2019). The flood management study explores the role of Western concepts of efficacy in Fijian cultural contexts; the relationship between self-efficacy, sources of efficacy, and flood protection behaviors in residents living in informal urban settlements in Fiji; and its application in South–North research. Protective behaviors are “any behavior performed by a person, regardless of the perceived or actual threat, to protect, promote, or maintain their life or livelihood, whether or not such behavior is objectively effective or not” (Harris and Guten 1979, p. 18).

#### Research context

The empirical research was conducted during the global coronavirus disease pandemic, which saw nationwide lockdowns and the cessation of global travel. To challenge Western knowledge hegemony, Fijian knowledge partners were recruited to be at the frontline of the flood management study to mitigate against the historical, political, and economic impact of European colonial rule and to generate culturally specific, rich data.

The pandemic meant that engagement with in-country knowledge partners was primarily limited to online-only modes, via video calls, emails, and messaging platforms. The time constraints and circumstances of the research meant that the benefits of remote research outweighed the negatives. However, the relational dynamics of online communication and engagement had its own challenges.

Issues like Internet lag disrupted conversations, hindering the normal flow of discussions. Technical constraints, such as camera placement, affected eye contact and gaze awareness, with some partners appearing disengaged and quieter partners communicating through others. Relying solely on visual and audio cues limited the capacity to interpret social and physical cues, potentially leading to misinterpretation. The observed distance between team members, both physically and metaphorically, impeded spontaneous discussions, limiting opportunities for relationship building and knowledge co-construction. These conditions restricted understanding of cultural norms and cues for engagement.

#### Data collection methods

The empirical participatory-observational research spanned September 2020–November 2022. Engagement and co-creation of research approaches was facilitated through presentations, role-play, scenario creations, discussions, and reflections. The lead author actively participated and observed the research process, from research socialization to training, through to data collection and analysis. Critical reflections enabled researchers to understand and observe the practical action of research and to turn the reflection inward to their own beliefs, assumptions, and behaviors in relation to the world around them (Mortari 2015). During the research process, the lead author systematically described and documented research events, social dynamics, attitudes, and behaviors relating to the knowledge co-construction between knowledge partners. Observations were recorded first-hand using qualitative methods (Angrosino 2007), including long- and short-form journal entries, contextual information, and reflections from the broader team obtained through WhatsApp messages. Analysis and discussion of preliminary research findings were explored online and in-person.

#### Data analysis method

Observations were described inductively and critically reflected upon throughout the research process. Themes and hypotheses were generated from these observations and provided vital insights that shaped the design and development of the systematic configurative review. These informed the development of the organizing framework presented in this paper.

### Systematic configurative review

Following challenging experiences in the empirical research, a systematic configurative review was undertaken to glean insights into effective South–North collaboration and practical engagement with diverse forms of knowledge. Conducted across Scopus and ProQuest databases in three stages (Feb 2021, March 2021, and May 2021), the review identified 74 articles for qualitative synthesis (see supplementary material for Literature search criteria and inclusion flow diagram).

The authors acknowledge the inherent limitation of focusing on Anglophone scientific articles and the potential bias introduced by excluding non-English-speaking cultures, posing a risk of perpetuating dominant Western perspectives the framework aims to overcome.

#### Data analysis method

Codes underwent thematic analysis within post-positivist constructivist and transformative paradigms, leading to a conceptual framework for operationalizing South–North knowledge construction (Braun and Clarke 2006; Kiger and Varpio 2020). The post-positivist constructivist paradigm posits that knowledge is socially constructed, and therefore, researchers’ and knowledge partners’ viewpoints are not separate from the research (Mertens 1998). Transformative research also acknowledges knowledge is socially situated, but it notes the influence of dominant or powerful groups on the construction and production of knowledge (Mertens 1998). It is through these lenses that the empirical cases within the literature were analyzed. Analysis included identifying and critically analyzing the context and actors and their power dynamics to determine the desired outcomes and the barriers and enablers of achieving these outcomes within the research process.

**Fig. 1:**
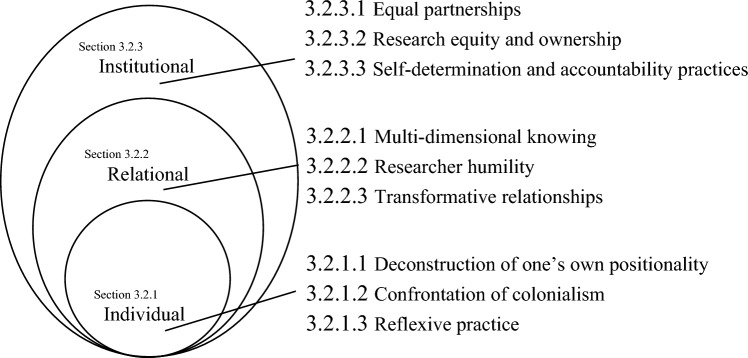
Nested South–North knowledge co-construction framework

Initial inductive coding of literature characteristics revealed 17 potential categories. Following further inductive analysis through descriptive empirical observations, these were organized into three interconnected levels: institutional, relational, and individual. These levels identified the decision-making context and sphere of influence within South–North knowledge co-construction. Within these contextual levels of influence, a total of nine guiding principles (see Fig. 1) and a subsequent 51 practical actions were identified (see Tables 1, 2, 3, 4, 5, 6, 7, 8 and 9), drawing on both insights from the systematic literature review and the authors’ empirical observations. These principles and actions are designed to enable researchers to engage with diverse forms of knowledge in South–North research and influence broader systemic change in the way knowledge co-construction is incorporated into research governance within academic institutions.

**Table 1:** Deconstruction of one’s own positionality

Putting it into practice: Deconstruction of one’s own positionality	Source
Deconstruction of one’s own positionality requires all knowledge partners to: Acknowledge researcher privilege by articulating and dissecting positionality Identify critical events or experiences that have shaped the way you understand and interact with the world, such as upbringing, cultural background, religious affinities, and academic, work, or personal experiences Identify what assumptions, biases, and preconceived notions or beliefs are implicit in your worldview and how you have approached the research Identify and acknowledge limitations in understanding expressed insights Expose yourself to diverse perspectives, opinions, and experiences and engage with literature in order to seek out information that challenges your existing views Engage in open and respectful conversations with people from different cultures and who hold different perspectives	Fiji case study Anthony-Stevens and Matsaw (2019), Castleden et al. (2017), Hart et al. (2017), LaVallie and Sasakamoose (2021), Lipscombe et al. (2021), Silva et al. (2020), Sylvester et al. (2020), Tilley (2017), Wilson et al. (2020)

**Table 2:** Confrontation of colonialism

Putting it into practice: confrontation of colonialism	Source
Confrontation of colonialism requires all knowledge partners to: Educate yourself on the design of colonial practices that seek to produce and reproduce power differentials Acknowledge the cultural histories, past and ongoing injustices and harm Question dominant beliefs, biases, language and assumptions Critically assess, dissect, and challenge western privilege and research production, extraction, power and commodification of knowledge Understand the broader social, economic, and political context of the research setting and power dynamics involved Examine the historical events and structural factors that have shaped, misrepresented or misinterpreted the worldviews of the country in which your research is being conducted and consider the ethical implications of how knowledge is portrayed Consult literature and critically reflect on which voices were prioritized, marginalized or omitted and consider how these factors influence knowledge co-construction	Fiji case study Adams et al. (2017), Anthony-Stevens and Matsaw (2019), Ebersöhn and Malan-Van Rooyen (2018), Lipscombe et al. (2021), Mazzetti and Blenkinsopp (2012), McNamara and Naepi (2018), Sylvester et al. (2020), Tilley (2017), Wilson et al. (2020)

**Table 3:** Reflexive practice

Putting it into practice: reflexive practice	Source
Reflexive practice requires all knowledge partners to: Listen actively, seek to understand diverse viewpoints, and be willing to question your own assumptions Actively and continually questioning research practices and assumptions and bringing this reflexivity to research practice Recognize diverse knowledge systems and expand one’s own worldview Engage with border forms of knowledge such as, cultural or implicit knowledge (traditions and societal norms), casual knowledge (connections between concepts or events), tacit and procedural forms of knowledge (those rooted in personal experience, intuition and context) Contribute to richer discussions and research approaches by acknowledging that different cultures, backgrounds and disciplines may have unique approaches to presenting and representing knowledge Continue to reflect on the knowledge shared to provide a more culturally sensitive understanding of diverse perspectives and the research context Continue self-reflection and understand that knowledge is dynamic and never an end point	Fiji case study Anthony-Stevens and Matsaw (2019), Hopkins et al. (2019), Lipscombe et al. (2021), McNamara and Naepi (2018), Silva et al. (2020)

**Table 4:** Multidimensional knowing

Putting it into practice: multidimensional knowing	Source
Multidimensional knowing requires all knowledge partners to: Recognize that colonial processes and Euro-American research norms are one form of knowledge and that all forms of knowledge are equally valuable. Counteract perspectives that science is position-less or objective reality by challenging binary notions of superior-inferior, valid and invalid forms of knowledge Investigate diversity of meaning and how knowledge is constructed, interpreted and experienced in a variety of ways by actively uncovering insights which may not be explicitly stated Acknowledge that not all knowledge systems are structured in the same way and that the relationality, dynamics, subtleties, or significance of what is shared cannot always be completely understood Overcome the perceived inflexibility of diverse knowledge systems through collective sense making and co-construction of shared meaning	Fiji case study Anthony-Stevens and Matsaw (2019), Castleden et al. (2017), Ebersöhn and Malan-Van Rooyen (2018), Hopkins et al. (2019), LaVallie and Sasakamoose (2021), Lipscombe et al. (2021), Matson et al. (2021), Mazzetti and Blenkinsopp (2012), Pham and Gothberg (2020), Raczek and Sugandhi (2020), Rai and Khawas (2019), Silva et al. (2020), Tilley (2017), van Meijl (2019), Wilson et al. (2020)

**Table 5:** Researcher humility

Putting it into practice: researcher humility	Source
Researcher humility requires all knowledge partners to: De-identify as the expert and place their perspective at the periphery. See other knowledge partners as equal contributors to knowledge co-construction Use open-ended questions to prompt and reflect on insights and experiences more deeply. Facilitate reflective discussions where meaning, significance and underlying insights can be explored Exercise patience and allow comprehension to evolve overtime. Adapt, refine or broaden research concepts or designs to ensure that approaches resonate with the complexity and specificity of the local environment	Fiji case study Anthony-Stevens and Matsaw (2019), Castleden et al. (2017), Day et al. (2020), Ebersöhn and Malan-Van Rooyen (2018), Frias-Navarro and Montoya-Restrepo (2020), Lipscombe et al. (2021), Matson et al. (2021), Raczek and Sugandhi (2020), Silva et al. (2020), Sylvester et al. (2020), Tilley (2017), van Meijl (2019)

**Table 6:** Transformative relationships

Putting it into practice: transformative relationships	Source
Transformative relationships requires all knowledge partners to: Provide and participate in formal and informal relationship building activities to explore new paradigms and experience each other's realities. Engage in the co-construction of research directions and agenda Collaborate in the collection of data, contextualization of insights and the development of research narratives Build trust through mutual respect and openness Build upon each other’s perspectives with empathy and understanding Be willing to adapt and collaborate	Fiji case study Adams et al. (2017), Anthony-Stevens and Matsaw (2019), Cameron et al. (2021), Castleden et al. (2017), Chambers (2007), Ebersöhn and Malan-Van Rooyen (2018), Hart et al. (2017), Hopkins et al. (2019), Lipscombe et al. (2021), Matson et al. (2021), Raczek and Sugandhi (2020), Wilson et al. (2020)

**Table 7:** Equal partnerships

Putting it into practice: equal partnerships	Source
Equal partnerships requires all knowledge partners to: Co-construct and agree on equal decision making authority in relation to research agendas, objectives and design Articulate and align on research roles and responsibilities Input and agree on a formal partnership agreement which considers governance and legal accountabilities and enables funding to be equally distributed between research partners Develop resource allocation, and funding policies Create ethics agreements and relationship management policies	Fiji case study Anthony-Stevens and Matsaw (2019), Beckford (2018), Hart et al. (2017), Hopkins et al. (2019), Kourantidou et al. (2020), Matson et al. (2021), Sylvester et al. (2020), Torso et al. (2020), Wilson et al. (2020)

**Table 8:** Research equity and ownership

Putting it into practice: research equity and ownership	Source
Research equity and ownership requires all knowledge partners to: Be cited as authors on publications and outputs. Equal access, management and ownership of data Equally participate and have authority over research inquiries, analysis, and dissemination Knowledge ownership, engagement and authority policies and protocols	Fiji case study Adams et al. (2017), Hopkins et al. (2019), LaVallie and Sasakamoose (2021), Matson et al. (2021), Pham and Gothberg (2020), Raczek and Sugandhi (2020), Sylvester et al. (2020), Tilley (2017), Torso et al. (2020), Wilson et al. (2020)

**Table 9:** Self-determination and accountability practices

Putting it into practice: self-determination and accountability practices	Source
Self-determination and accountability practices requires all knowledge partners to: Develop a research agreement which details the equitable distribution and involvement in research outputs, including the research direction, co-construction and evaluation processes, checkpoints, milestones and escalation points Equal access to knowledge, research resources, and funding Create a self-determination and accountability policy that provides clear standards and expectations, and that all parties can be held accountable Establish consensus building and decision-making policies and protocols Develop research and partnership evaluation and accountability protocol in line with the research agreement	Fiji case study Beveridge et al. (2020), Hopkins et al. (2019), Kourantidou et al. (2020), LaVallie and Sasakamoose (2021), Lipscombe et al. (2021), Matson et al. (2021), McNamara and Naepi (2018), Pham and Gothberg (2020), Raczek and Sugandhi (2020), Sylvester et al. (2020), Tilley (2017), Torso et al. (2020), Wilson et al. (2020)

## Results and findings

### Framework architecture

The organizing framework presented in this paper aims to help researchers working across geographies navigate, understand, and engage with diverse forms of knowledge into interdisciplinary South–North research (see Fig. 1). The framework is represented as three nested circles, designed to depict the interdependencies and influence loops in knowledge co-construction. Knowledge co-construction may be addressed at a particular level or simultaneously across levels, influencing one another. The authors hope the framework addresses the well-founded critiques of Western-centric research approaches, satisfies the demands of South–North research, and offers research foundations for further exploration and framework development.

This first part of this article presents the core framework elements: the contextual levels of influence. The second part uses these levels to articulate the ways in which researchers can navigate through these contexts and includes guiding principles and practical actions for integrating diverse forms of knowledge into South–North research.

#### Three nested contextual levels of influence

The empirical cases in the literature revealed the negative impact of the hegemonic and colonial nature of research (Broesch et al. 2020). Historic academic assumptions of the universality of knowledge were attributed to the perception that there is a knowable reality to be extracted (Gone 2018; Kotze and Dymitrow 2021; Schmidt and Pröpper 2017; Windchief et al. 2017). This perception was a core theme depicted in the literature and was observed in the subsequent research practices that empirical cases sought to overcome (Anthony-Stevens and Matsaw 2019; Brandt et al. 2013; Dentoni et al. 2018; Hopkins et al. 2019; Jentsch 2004; KFPE 2018; Kotze and Dymitrow 2021; Lodge and Wegrich 2014; O’Flaherty et al. 2008; R’boul 2020; Sastry and Ramasubramanian 2020; Schmidt and Pröpper 2017; Smith 2012; Waddell et al. 2015; Windchief et al. 2017).

Empirical observations of the Fiji case study and the empirical cases reviewed in the literature revealed three key levels of South–North knowledge co-construction: institutional, relational, and individual. The institutional level focuses on academic institutions and the formal and informal norms and rules that shape research practices (Anthony-Stevens 2020; Beveridge et al. 2020; Bremer et al. 2021; Cameron et al. 2021; Lipscombe et al. 2021). The relational level refers to the interaction, interpretation, and influence of two or more actors within a research project or program (Anthony-Stevens 2020; Castleden et al. 2017; Ebersöhn and Malan-Van Rooyen 2018; LaVallie and Sasakamoose 2021; Windchief et al. 2017). The individual level relates to each researcher’s attitudes, beliefs, and assumptions within their sphere of influence (Anthony-Stevens 2020; Ebersöhn and Malan-Van Rooyen 2018; LaVallie and Sasakamoose 2021; Pham and Gothberg 2020; Sylvester et al. 2020). Within each contextual level of influence, three guiding principles enable researchers to engage with diverse forms of knowledge.

#### Guiding principles

The empirical cases reviewed in the literature revealed many ways to engage with diverse forms of knowledge. Seminal contributions included participatory action research, community-based participatory research, and decolonial and Indigenous methodologies, such as “Two-Eyed Seeing” (Iwama et al. 2009). Most empirical cases focused on integrating local and traditional knowledge. Predominantly, these cases featured in-person collaboration between international researchers and local or traditional knowledge partners, in the same country. Notably, core themes featured in the empirical cases, such as collaboration, empowerment, and joint decision-making (Dentoni et al. 2018; Jentsch 2004; Waddell et al. 2015), can and were applied to knowledge co-construction across geographies and, specifically, in the Fiji case study. The lack of sufficient articulation of *how* to effectively incorporate diverse viewpoints and knowledge (Kotze and Dymitrow 2021; Schmidt and Pröpper 2017) led to this paper’s authors critically analyzing the research context, the actors, their assumptions and biases, and the power dynamics within the published empirical cases—to develop nine guiding principles to help researchers navigate, interpret and engage with diverse ways of knowing. These principles include “Deconstruction of one’s own positionality”, “Confrontation of colonialism”, “Reflexive practice”, “Multidimensional knowing”, “Researcher humility”, “Transformative relationships”, “Equal partnerships”, “Research equity and ownership”, and “Self-determination and accountability practices”.

#### Practical actions/steps

Empirical cases noted in the literature revealed numerous practical actions researchers could take to understand and engage with diverse forms of knowing. However, most actions related to in-person relationship building, through the creation of safe spaces, or spending time in communities (Hopkins et al. 2019; McNamara and Naepi 2018; Windchief et al. 2017). Observations from the Fiji case study revealed the inadequacies of existing positivist research practices. Limited historical and cultural understanding, and fixed definitions of knowledge and existing processes and procedures, were noted as limitations in knowledge (Anthony-Stevens and Matsaw 2019), for they unintentionally reproduced existing Western-centric views of knowledge (Castleden et al. 2017). Inductive reasoning regarding these barriers and enablers led to the development of a list of practical actions researchers can take to engage with diverse viewpoints and ways of knowing. These actions are presented in “Deconstruction of one’s own positionality”, “Confrontation of colonialism”, “Reflexive practice”, “Multidimensional knowing”, “Researcher humility”, “Transformative relationships”, “Equal partnerships”, “Research equity and ownership”, and “Self-determination and accountability practices”.

### Framework concepts

The key concepts within the organizing framework are presented in the following section.

#### Individual level

The individual level refers to conscious and subconscious ways in which knowledge is generated and subsequently viewed and valued (Anthony-Stevens 2020; Bozhkov et al. 2020). It relates to the beliefs, assumptions, cognitive biases, and heuristics that underpin the way researchers approach, interpret, and engage with knowledge (Anthony-Stevens 2020). Empirical cases reveal the prevalent assumption that Western knowledge is universal, hindering knowledge co-construction and fostering implicit bias that ‘Other’ forms of knowledge are inferior (McNamara and Naepi 2018, p. 343). For instance, Bozhkov et al.’s (2020) study exposes researchers’ misconception about “helping to *teach* Indigenous First Nation people,” highlighting a lack of recognition for Indigenous knowledge and their assumption that “Indigenous people want or need to be “taught” (Bozhkov et al. 2020, p. 231). This example illustrates how individual notions, shaped by dominant views cloud the way researchers approach and engage with diverse forms of knowledge.

Similarly to the efforts of many scholars (Anthony-Stevens 2020; Bozhkov et al. 2020; LaVallie and Sasakamoose 2021; Lipscombe et al. 2021; McNamara and Naepi 2018), the framework is an effort to articulate how researchers can critically assess and challenge their own attitudes, beliefs, and assumptions in order to deconstruct the colonial viewpoint in which scientific knowledge has historically been positioned (Adams et al. 2017). The individual level within the conceptual theoretical framework has three guiding principles: Deconstruction of one’s own positionality, Confrontation of colonialism, and Reflexive practice.

##### Deconstruction of one’s own positionality

Attitudes, beliefs, and values are shaped by the individual context and societal, environmental, and cultural backgrounds (Castleden et al. 2017). Empirical cases in the literature highlighted the importance of questioning one’s own attitudes and beliefs to understand how individuals see the world (Adams et al. 2017; Anthony-Stevens and Matsaw 2019; Caretta and Jokinen 2016; Rydgren 2011; Sylvester et al. 2020). McNamara and Naepi (2018) have noted that the perception that Western knowledge is universal, whether “benevolent or hostile,” creates binary assumptions about what knowledge is valid, critically affecting the way diverse forms of knowledge are represented in research (McNamara and Naepi 2018, p. 343).

Researchers must explore and dismantle the factors that shaped their beliefs in order to confront them (Rydgren 2011). Effective deconstruction relies on researchers being able to question, understand, assess, and articulate the factors that influenced their beliefs. and become open to identifying their biases, beliefs, and assumptions and questioning them (Lipscombe et al. 2021).

Findings from the Fiji case study revealed that deconstruction starts with identifying the factors that shape one’s own worldview. This process started with identifying the cultural and social systems which influence one’s beliefs, assumptions and biases, such as an individual's upbringing, cultural background, religious affinities, academic or work experiences and resulted in reflection on the validity of these beliefs.

By identifying the foundations of one’s knowledge, researchers in the Fiji case study became aware of the impacts of binary assumptions of valid knowledge and enabled ‘normative’ language such as ‘training’ to be challenged and changed. Initially perceived as neutral, reflection revealed its embodiment of top-down hegemonic views of what is ‘true’ or ‘false’—which reinforced the dominant belief that knowledge is universal. Unpacking structures and systems that reinforce specific ways of knowing (Pham and Gothberg 2020) enabled the identification of factors that had shaped these beliefs and allowed assumptions to be acknowledged and countered. For instance, binary training methods, such as multiple-choice quizzes, hindered both the understanding of diverse perspectives and the co-construction of knowledge by neglecting the Fijian context. Challenging such perceptions, as shown in empirical cases, enables researchers to change the way they interact and view the world around them, facilitating more effective assessment of convergences or divergences in diverse knowledge systems (Hopkins et al. 2019). This understanding deepens researchers’ insights into how diverse forms of knowledge have been developed.

Consistent with empirical literature, the Fiji case study found that the deconstruction of one’s knowledge system can be aided through comparing it to other ways of knowing and historical, cultural, and social systems (Adams et al. 2017; Sylvester et al. 2020; Wilson et al. 2020). This was conducted through exposure to diverse perspectives, opinions, and experiences that challenge existing views (see Table 1 for a summary of the practical steps, derived from insights from the Fiji case study and empirical studies in literature).

##### Confrontation of colonialism

Colonial philosophies of domination and superiority, under the guise of neutrality, are foundational to Western beliefs and biases (Wilson et al. 2020). Some studies have highlighted the importance of interrogating privilege and the historic and ongoing colonialism in order to subjugate cultural biases limiting the co-construction of South–North knowledge (Adams et al. 2017; Anthony-Stevens and Matsaw 2019; Tilley 2017). Further, it is essential to learn the historic effects of colonialism to understand and deconstruct one’s own perspective (Adams et al. 2017; Lipscombe et al. 2021; Sylvester et al. 2020). Through comprehending and acknowledging the concepts underlying colonialism, researchers may gain invaluable experience of the ways in which their knowledge systems limit their acceptance of other forms of knowledge (Lipscombe et al. 2021).

Empirical insights from the Fiji case study revealed the importance of examining the historical events and structural factors that contributed to shaping one’s worldview and understanding the broader social, economic, and political context on research settings. This process enables the researchers to ask critical questions about the systems and structures that shape their worldviews and the historical context and power dynamics involved.

One way that colonialism occurs is through the language and framing within Western research. Terms such as participants, respondents, and subjects perpetuate the perception that there is a universal knowledge to be extracted (Windchief et al. 2017). Lack of cultural and historical understanding of colonialism and the way it is perpetuated is a common barrier to knowledge co-construction and perpetuates power differences and top-down notions of empowerment (Bozhkov et al. 2020).

The empirical cases in the literature revealed the critical need for researchers to educate themselves on the design of colonial practices that seek to produce and reproduce power differentials (Adams et al. 2017; McNamara and Naepi 2018).

The Fiji case study saw researchers critically reflect on which voices were prioritized, marginalized or omitted and consider how these factors influenced the research approach and knowledge co-construction. Their examination of existing literature also enabled the researchers to question the framing of information, identify underlying assumptions, and evaluate the credibility of sources to foster a more discerning approach to knowledge co-construction and cross-cultural research.

Further, empirical observations and reflections from the authors’ Fiji case study found power differentials also reinforced historical and ongoing colonialism. While the research was socialized with Fijian knowledge partners, the interpretation of literature and the generation of methodological approaches were confined to Western knowledge partners. In the Fiji case study, findings from the literature review and methodological approach were presented with the intention of Fijian knowledge partners leading and shaping the research approach. Although socialization included empowerment techniques, such as collaborative workshops and brainstorming sessions, the lead authors' lack of understanding of how underlying colonialism presents itself in different knowledge presentation styles inhibited knowledge co-construction. The presentation of literature review findings and theoretical models unintentionally portrayed the approach as finalized. This, paired with the lack of early collaboration, unintentionally enforced paternalistic collaboration forms, subconsciously defining the roles of Fijian knowledge partners as those enacting the research and of Western knowledge partners as those defining it.

In line with Sylvester et al.’s (2020) findings, empirical observations from the Fiji case study revealed that through comprehending and acknowledging the underlying concepts of colonialism, researchers can gain awareness of these taught “truths” and develop invaluable experience of the ways in which their knowledge systems limit their acceptance and subsequent engagement with other forms of knowledge (see Table 2 for a summary of the practical steps).

##### Reflexive practice

Reflexive practice does not seek to replace one dominant form of knowledge with another (Castleden et al. 2017). It enables researchers to realize Western knowledge is not universal or superior to other forms of knowing but, rather, challenges and broadens existing views of knowledge (Lipscombe et al. 2021; McNamara and Naepi 2018; Silva et al. 2020). Through disengaging from these limiting attitudes and beliefs, researchers can fully situate themselves in the cultural context as learners and build equal relationships with knowledge partners (Lipscombe et al. 2021).

According to Hopkins et al. (2019) and LaVallie and Sasakamoose (2021), when individuals are reflexive, they seek to critically assess how perspectives are formed, which helps them limit incongruence from ‘other’ forms of knowing. Empirical observations from the authors’ Fiji case study highlighted the ongoing nature of actively and continually questioning assumptions and bringing this reflexivity to research practice. This finding is consistent with Anthony-Stevens and Matsaw’s (2019) view that knowledge formation is a never-ending process.

In the authors’ Fiji case study, theoretical concepts and frameworks relating to self-efficacy were core to the research approach. Discussions surrounding the concept of self-efficacy with Fijian colleagues revealed that self-efficacy was seen as indistinguishable from confidence and response efficacy. While conceptual blurring was initially met with further clarification of concepts and theoretical differences, further discussions highlighted the need for concepts to be discussed and explored from a Fijian perspective. Active listening ascertained that the concept of self-efficacy was less applicable in the Fijian research context due to Fiji’s collectivist culture. Joint reflection and discussions surrounding collectivism and the informality of research communities (urban informal settlements) highlighted the contextual need for residents to work together. Resulting in the broadening of concepts and terminology to reflect cultural realities.

The process of identifying and reflecting on knowledge provided a more nuanced, critical, and culturally sensitive understanding of the research context, and emphasized the importance of acknowledging diverse perspectives and recognizing the contextual nature of knowledge. Through this process, a critical limitation of the initial literature review and methodological approach was also identified. The focus on academic literature unintentionally limited the exploration of diverse knowledge types for it failed to incorporate broader forms of knowledge such as cultural or implicit knowledge (traditions and societal norms), casual knowledge (connections between concepts or events), and tacit and procedural forms of knowledge (those rooted in personal experience, intuition, and context).

Similar to the study of Ebersöhn and Malan-Van Rooyen (2018), in the authors’ Fiji case study, the research team found that the process of individually and jointly identifying and reflecting on how knowledge is presented and represented helped to deconstruct what it means to know. This process of reflection involves articulating the context in which knowledge is derived to examine and unpack the factors that have shaped its representation.

In the Fiji case study, this meant acknowledging that the concepts of self-efficacy originated from Western Academia and must be applied differently in individualistic and collective cultures. The process of reflecting on how context knowledge is represented enabled the research team to gain greater awareness and understanding that knowledge is not neutral and is often influenced by various factors. This reflection identified that knowledge produced within a specific cultural context is not universally applicable and helped the research team to explore the complexities and nuances within a given subject, moving beyond simplistic or polarized perspectives and adapting the theoretical frameworks to be more culturally relevant (see Table 3 for a summary of the practical steps).

#### Relational level

The relational level within this framework refers to knowledge co-construction through the interaction of two or more actors. To do this, knowledge partners need to understand knowledge is connected to, and nested within, cultural and contextual settings and is co-constructed through social interaction, interpretation, and shared meaning making (Anthony-Stevens and Matsaw 2019; Hall and Callery 2001; Lipscombe et al. 2021; Rai and Khawas 2019; Spretnak 2011). The relational level within the organizing framework has three guiding principles: multidimensional knowing, researcher humility, and transformative relationships.

##### Multidimensional knowing

The universality of Western knowledge has historically resulted in the rejection of “other” knowledge systems and has limited the effectiveness of South–North collaboration (Adams et al. 2017; McNamara and Naepi 2018). A multidimensional approach to South–North knowledge co-construction plays a critical role in reducing universal perceptions of knowledge and creates room for multiple forms of knowing. Multidimensional knowledge requires researchers to place their perspective at the periphery and see knowledge partners as equal contributors to knowledge and knowledge co-construction (Castleden et al. 2017).

Although many collaboration techniques were used in the Fiji case study to generate shared meaning making, such as group discussions, role play, reflections, and scenario creation activities, not all techniques generated the desired research critiques and constructions. The presentation of theoretical descriptions of knowledge lacked the contextual application to lived experiences, resulting in limited discussions and sense making, resulting in local knowledge partners leaning away from, rather than into, group discussions and role-playing activities. Conversely, collaboration techniques, such as personal reflections and scenario creation activities, revealed key insights into diverse forms of knowledge systems and ways of knowing. However, experiences from the Fiji case study revealed that international knowledge partners need to go further than recognizing and acknowledging these differences. They must facilitate knowledge co-construction through actively uncovering insights which may not be explicitly stated. The Fiji case study revealed the effectiveness of encouraging individuals to share stories as they helped to reveal implicit knowledge embedded in the details and nuances of personal accounts, as well as the ability for group discussions to build on individual reflections and uncover deeper understanding.

In the Fiji case study, a pilot study tested the research approach's effectiveness by questioning heads of households on flood protection. Data from the pilot study (interviews and photographs) and reflections validated knowledge partners perspectives, revealing residents linked individual efficacy to family or group efforts. Without upfront discussions on the collective nature of communities, social dynamics and factors influencing residents' efficacy, critical insights might have been dismissed, missing crucial insights into community social dynamics for flood resilience development. These findings underscore the crucial role for exploring shared knowledge deeply in the early stages of research design. Consistency with empirical cases in the literature, the Fiji case study found that multidimensional knowing requires questioning, building upon perceptions, and holding multiple viewpoints simultaneously (Wilson 2008; Windchief et al. 2017) (refer to Table 4 for a summary of practical steps).

##### Researcher humility

Knowledge co-construction requires humility and the acceptance from knowledge partners to listen openly and engage with each other without objectification or binarism (Lipscombe et al. 2021; Silva et al. 2020). Empirical cases in the literature revealed the need to challenge knowledge hierarchies, which perpetuate the notion of superiority, toward facilitating effective knowledge co-construction (Anthony-Stevens and Matsaw 2019). Further, researchers need to set aside their ingrained perceptions of universality and super-inferior beliefs and practices to detach from authority and replace it with humility (Adams et al. 2017).

Researcher humility requires abandoning the perception of being experts and, instead, believing that all knowledge partners have skills and knowledge to contribute (Anthony-Stevens and Matsaw 2019; Castleden et al. 2017; Ebersöhn and Malan-Van Rooyen 2018; Matson et al. 2021; Mazzetti and Blenkinsopp 2012; Wilson et al. 2020). To listen and provide the space and time to explore, articulate, and interpret diverse perspectives, without expectation that concepts can be understood within a Western framework (Anthony-Stevens and Matsaw 2019; Castleden et al. 2017; Matson et al. 2021; Rai and Khawas 2019).

Although this was true in the Fiji case study, the extent to which it was incorporated was constrained by the flow-over effects of subtle dynamics from preexisting projects in the parent program, which required different forms of partnership. However, through iteratively questioning and embracing contributions from local knowledge partners, ‘other’ forms of meaning and knowing were investigated, strengthening the research. This process involved acknowledging and engaging with insights from various sources. Facilitating reflective discussions where meaning, significance and underlying insights were explored and using open-ended questions to prompt and reflect on insights and experiences more deeply. This process served to narrow the divide between the existing research theory and the cultural theories and realities that may not be represented in academic journals or literature.

Through immersion in cultural contexts and engagement with knowledge holders, researchers reevaluated existing theoretical concepts and prompted modifications to the research design and a reassessment of assumptions, strengthening research outcomes. This adaptation ensured the theoretical framework aligned with the complexity of the local environment, unveiling more contextual and meaningful insights.

Revisiting local partner insights during analysis allowed researchers to contextualize findings. The Fiji case study uncovered the significance of traditional village hierarchy and kinship structures deeply ingrained in community social fabric. The common lineage fostered strong social cohesion, and hereditary roles provided clear leadership in flood exposure and recovery. Understanding the importance of ‘Matavuvale’ (extended family) helped differentiate between family efficacy and collective efficacy in flood responses. This nuanced outcome resulted from ongoing engagement with Fiji knowledge partners—highlighting the continuous nature of knowledge co-construction and the need for researcher humility at various stages (Anthony-Stevens and Matsaw 2019; Castleden et al. 2017) (see Table 5 for a summary of the practical steps).

##### Transformative relationships

Transformative relationships, grounded in respect and collective learning (Hopkins et al. 2019; Torso et al. 2020), are cultivated through both formal and informal research engagement (Raczek and Sugandhi 2020; Silva et al. 2020; Torso et al. 2020; Wilson et al. 2020). Formal engagement involves co-constructing research directions, collaborating on data collection, contextualizing insights, and developing narratives (Castleden et al. 2017; Raczek and Sugandhi 2020; Tilley 2017; Wilson et al. 2020). Informal engagement includes participating in local social activities, cultural gatherings, and religious ceremonies (Hopkins et al. 2019), offering knowledge partners opportunities to experience diverse perspectives (Raczek and Sugandhi 2020; Silva et al. 2020; Torso et al. 2020; Wilson et al. 2020). While international researchers often engage with local communities informally, there is a notable disparity, with limited instances of local communities or knowledge partners gaining insights into the lives of international researchers.

Knowledge co-construction is developed through equitable, trusting research partnerships, whereby meaning is situated and negotiated within cultural and social systems and contextual interactions (Castleden et al. 2017; LaVallie and Sasakamoose 2021). Unlike the empirical cases in the literature, the Fiji case study was conducted remotely, as part of a doctoral research project, limiting the level of co-construction. Although not the desired approach, research relationships were built online through one-on-one and group calls on Zoom, emails, and WhatsApp chats. The use of both formal and informal channels provided knowledge partners the opportunity to collaborate on research approaches and to share not only research reflections and progress updates but also aspects of their personal lives and experiences. Through these forms of engagement, trust, understanding, and shared meaning was built which, in turn, strengthened their research relationships. The teams focus on mutual respect and openness saw members value and build upon each other’s perspectives. Empathy and understanding increased knowledge partners' comfort in sharing their thoughts, ideas, and concerns. This in turn enabled potential research challenges to be mitigated quickly and an increase in the overall willingness to adapt and collaborate.

While face-to-face and situated engagement would have built more robust relationships and enhanced knowledge co-construction, the lead author’s prior visits to the research communities aided relationship development and contextual understanding during data collection. Post data collection meetings in Melbourne allowed knowledge partners to collaborate on interim findings, highlighting the strength and equitable relationships formed during the research (Castleden et al. 2017; Chambers 2007; Ebersöhn and Malan-Van Rooyen 2018; Hart et al. 2017; LaVallie and Sasakamoose 2021). In contrast to the start of the research collaboration, where existing dynamics reinforced top-down structures, power dynamics achieved equilibrium over the study’s duration. This was illustrated by members engaging in open discussions, decision making and expressing shared ownership in the research. This experience underscores the benefits of face-to-face relationship building and the added depth body language brings to meaning making (see Table 6 for a summary of the practical steps).

#### Institutional level

The institutional level in this framework refers to academic institutions and the formal and informal norms and rules that shape behavior and influence South–North knowledge co-construction (Bremer et al. 2021). Norms and rules are designed to aid an institution’s effective functioning and prescribe the appropriate behavior or actions within it (Alexandra 2016; Bremer et al. 2021; Harris et al. 2010). Exploration and reflection of these guiding principles ensure these norms and rules are fair and ethical. To aid South–North knowledge co-construction, there are three key principles, containing practical actions: equal partnerships, research equity and ownership, and self-determination and accountability policies.

##### Equal partnerships

Literature has long noted the importance of partnerships (Dentoni et al. 2018; Waddell et al. 2015; Waddock et al. 2015). Historically, South–North research collaborations have been formed through research partnerships negotiated and facilitated by Western institutions (Beveridge et al. 2020; Sylvester et al. 2020; Wilson et al. 2020). Research funders formalize these partnerships by requiring a lead institute for funding applications (Sylvester et al. 2020; Wilson et al. 2020). This tends to result in the unequal distribution of funding and accountability to lead institutions, which limits the equitable distribution of power and resources between research partners (Beveridge et al. 2020; Sylvester et al. 2020; Wilson et al. 2020). It results in South–North research being unequally directed by lead academic institutions, thus reinforcing colonialism through paternalistic concepts of knowledge sharing and extraction (Sylvester et al. 2020).

According to Beveridge et al. (2020), although research institutions have promised to provide equal decision-making authority to knowledge partners, the fundamental structure of academic institutions means ongoing colonialism is embedded at their core. Empirical cases in the literature indicate that governance and legal accountabilities restrict the inclusion of equal knowledge partners (Sylvester et al. 2020). However, the ingrained, ‘subtle’ nature of institutions’ Western-centric bias is another complexity researchers need to overcome.

Anthony-Stevens (2020) found that “the majority of researcher students interviewed struggled to understand and unpack the colonial structures, which supported and reinforced inequitable partnerships.” This is also true of the authors’ Fiji case study, in which concepts of empowerment and collaboration were initially considered “effective” methods for knowledge sharing and co-construction. Reflections during the Fiji case study, revealed that top-down colonial forms of engagement often reinforce unequal power dynamics and perpetuate a Western-centric bias, such as limited decision-making input and leadership opportunities, inequitable control over critical resources or funding, and academic hegemony, whereby Western knowledge is prioritized over Indigenous or other forms of knowledge.

As noted by Cameron et al. (2021) and Hopkins et al. (2019), these inequitable forms of partnership perpetuate the unequal distribution of power between knowledge partners (Beveridge et al. 2020; Cameron et al. 2021; McNamara and Naepi 2018). Collaborative efforts should prioritize equitable power distribution to address colonialism.

Dynamic interactions and collective meaning making require cooperation, reciprocation, common objectives, and trust (Goswami and Agrawal 2019; Holste and Fields 2010). Equal partnerships enable research agendas and outcomes to be developed with knowledge partners and within the environments for which they are intended (Cameron et al. 2021; Hopkins et al. 2019). Through the formalization of equal partnerships, all parties detail, and commit to sustain and nurture, the equitable distribution of power and resources across the research process and provide iterative feedback and evaluations to aid the adjustment and maintenance of South–North knowledge partnerships (Matson et al. 2021) (see Table 7 for a summary of the practical steps).

##### Research equity and ownership

Effective South–North knowledge co-construction requires equitable distribution of power and resources (Hopkins et al. 2019). Equitable ownership and research relationships democratize and decolonize research and limit conscious or unconscious paternalistic norms and concepts of knowledge co-construction (Beveridge et al. 2020; Cameron et al. 2021; McNamara and Naepi 2018; Sylvester et al. 2020). It enables knowledge partners to co-own research data and have intellectual equality over the research (Torso et al. 2020). Empirical cases in the literature referenced the need to establish negotiations at the commencement of research and detailed in ethics applications, institutional performance metrics, and funding models (Sylvester et al. 2020; Torso et al. 2020; Wilson et al. 2020). This upfront agreement creates and sustains a safe space and limits top-down paternalism and the exclusion of knowledge partners from decision-making, analysis, and knowledge co-construction.

In addition, empirical cases in the literature have outlined the importance of recognizing and including knowledge partners on research outputs (LaVallie and Sasakamoose 2021; Lipscombe et al. 2021; Raczek and Sugandhi 2020; Torso et al. 2020). Counter-viewpoints suggest that recognition alone is tokenistic for it fails to create knowledge equity (Hart et al. 2017; Tilley 2017). Existing agreements and ownership structures within the parent project, and institutional doctorate requirements, meant the mechanisms relating to research equity and ownership were limited.

The lead author acknowledges that ensuring research equity and ownership should be emphasized in addition to recognition and inclusion (Hopkins et al. 2019; Raczek and Sugandhi 2020; Tilley 2017), but existing institutional structures inhibited research equity and co-ownership in the Fiji case study. This fact highlights the complexity and challenges in relation to effecting change at the institutional level (see Table 8 for a summary of the practical steps).

##### Self-determination and accountability policies

Self-determination requires knowledge partners to have the autonomy, ability, and authority to shape the direction of research (Gagne and Deci 2005). Unlike informal or unspoken research practices and expectations, self-determination and accountability policies ensure that they have the ability to outline clear standards and expectations, and that all parties can be held accountable (Matson et al. 2021). According to the empirical cases in the literature, formalizing self-determination and accountability practices ensures knowledge partners can and are intrinsically motivated to determine whether research directions are relevant and whether research processes are designed and implemented to prevent cultural misinterpretation of research data (LaVallie and Sasakamoose 2021; McNamara and Naepi 2018). It is crucial that knowledge partners have self-determination over research decisions in order to prevent knowledge being extracted and codified without consent (Beveridge et al. 2020; Kourantidou et al. 2020; LaVallie and Sasakamoose 2021; Tilley 2017).

Moreover, self-determination includes equal access to knowledge and research resources, along with active involvement in research discussions (Hopkins et al. 2019; LaVallie and Sasakamoose 2021). Literature suggests that institutional policies should allow research partners to challenge universal knowledge constructs, promoting the value of all knowledge forms (Castleden et al. 2017; Ebersöhn and Malan-Van Rooyen 2018). However, historical inequalities in access have limited knowledge partners' capacity and authority to co-construct knowledge (LaVallie and Sasakamoose 2021; Tilley 2017; Wilson et al. 2020).

In the Fiji case study, existing funding stipulations and institutional practices unintentionally reinforced superior–inferior perceptions, limiting Fiji knowledge partners' autonomy. The study's failure to incorporate self-determination and accountability policies restricted how knowledge forms were presented and incorporated, leading to ambiguity about knowledge partners' roles. Observations align with literature and suggest that formalizing these policies would clarify roles, fostering more equitable knowledge co-construction (Anthony-Stevens and Matsaw 2019; Ebersöhn and Malan-Van Rooyen 2018; Mazzetti and Blenkinsopp 2012) (refer to Table 9 for a summary of practical steps).

## Conclusion

In the domain of interdisciplinary South–North research, scholars must critically assess dominant research approaches and knowledge production, recognizing their potential to reproduce, control or co-construct knowledge forms. The nested Knowledge Co-construction Framework, presented in this paper, builds on seminal studies to illustrate the interdependence and influence in the co-construction of knowledge, acknowledging how the research context can positively or negatively shape this process. The framework provides guiding principles and practical actions for navigating South–North research, emphasizing that knowledge co-construction can occur at specific levels or simultaneously across multiple levels, each influencing the others.

While instrumental in guiding interdisciplinary South–North research practice, the Nested Knowledge Co-construction Framework does not address the systemic barriers to knowledge co-construction in academia. While it emphasizes the need for researchers and institutions to proactively mitigate colonial bias and engage with local knowledge partners, it does not seek to provide an exhaustive exploration of broader challenges, such as the pervasive influence of a neoliberal structure on researchers (Billot 2010; Kidman 2020). Prioritizing metrics like publication counts and citation indices, diminishing public funding, commercialization of research, and shifts toward economic competitiveness limit opportunity for knowledge co-construction. Comprehensive attention to aspects like time, community, fair compensation, and active engagement—essential for fostering trust and relationships—require institutional change and augmented funding.

These considerations extend to all researchers, including early career researchers navigating an academic system entwined with colonial legacies and neoliberal ideals. As we aspire to test and refine this framework, reflection on these broader challenges inherent in academia is crucial. Doing so enhances the framework's applicability within the complex landscape of academic research, fostering more nuanced and effective knowledge co-construction practices.

This framework reflects the authors’ personal and shared journey of knowledge co-construction. Their hope is that the framework will strengthen research approaches and its application will be tested in various settings to evaluate its utility and effectiveness, and to further develop its foundations.

### Supplementary Information

Below is the link to the electronic supplementary material.

Supplementary file1 (DOCX 41 KB)
