# Supplementary material for: An organizing framework to break down Western-centric views of knowledge in North–South research
Source: Sustain Sci. 2024 Feb 17;19(2):647–64. doi: 10.1007/s11625-024-01478-6 (PMC10891260; doi:10.1007/s11625-024-01478-6)
Supplement: Supplementary file 1 — Supplementary file1 (DOCX 41 KB) [file 11625_2024_1478_MOESM1_ESM.docx]

**Supplementary material**

*Data collection method*

Studies featured in the systematic review were broad in scope, used varying terminologies and had little consensus in findings. Characteristics of South–North knowledge construction were extracted from the literature using inductive reasoning. Extracted characteristics included empirical, participatory-observational, subjective, and experiential information and were coded as barriers and enablers. These specific characteristics were then aggregated and thematically coded to generate general conclusions in relation to South–North knowledge co-construction.

The systematic review was conducted in three stages across two databases, Scopus and ProQuest, during Feb 2021, March 2021, and May 2021. The initial searches failed to uncover an existing operational framework and revealed limited empirical studies on knowledge co-construction in South–North research. These initial findings resulted in the search being broadened to focus on cross-cultural research contexts and include theoretical studies and the dominant terminology used across disciplines (see Table 1).

Certain terms, such as “learning” and “co-management of knowledge,” were found to skew the results toward education, healthcare, and business contexts. Further thematic analysis identified search terms, subjects, and journals for exclusion, and revealed two key questions to be asked of the literature: (a) whether the study involved cross-cultural collaboration, and (b) whether any ethical implications limited knowledge co-construction, such as power differentials seen in education, employment, or healthcare contexts.

The final search criteria incorporated learnings from the first two stages (see Table 1). Owing to the breadth of results from earlier iterations and the infancy of the research topic, the final search criteria focused on studies in social sciences, environmental sciences, and psychology; multidisciplinary studies, and studies published in the past five years.

**Table 1 Literature search criteria**

| Terms related to knowledge | “Knowledge translation" OR "knowledge transfer" OR "knowledge framework" OR "knowledge exchange" OR "knowledge uptake" OR "knowledge linkage'" OR "ways of knowing" OR "legitimate knowledge" OR "knowledge production" OR  "knowledge systems" OR "knowledge processes" OR "knowledge representations" OR "ways of knowing that" OR "ways of knowing how" OR "ways of coming to know" OR "different epistemologies" OR "different knowledge systems" OR "forms of knowledge" OR "integrated knowledge" OR "associated knowledge systems" OR "ways of being" OR "co-management of knowledge" OR "collaborative research" |
| --- | --- |
| **AND** Terms related to western knowledge | "Western knowledge" OR "decolonization" OR "western ethnocentrism" |
| **AND** Terms related to other forms of knowledge | "Traditional knowledge" OR "cultural knowledge" OR "local knowledge" OR "boundary work" OR "boundary crossing" OR "boundary practice" |
| **AND** Terms related to cross-cultural knowledge | "Cultural conceptualizations" OR "cross-cultural ways of knowing" OR "intra-cultural ways of knowing" OR "inter-cultural ways of knowing" OR "cultural translation of knowledge" OR "propositional knowledge" OR "contextual knowing" OR "self-concepts" OR "world views" OR "perspectives" OR "cultural capital" OR "cultural plurality" |

The review, conducted across Scopus and ProQuest databases, resulted in 283 empirical and theoretical papers. Titles and abstracts were screened and assessed against the search criteria and duplicate and unavailable records were removed. A total of 137 articles were read in full, and a further 63 articles were removed. Articles that did not include South–North knowledge or where ethical implications limited knowledge co-construction, were removed. A total of 74 articles were included in the qualitative synthesis. Fig. 1 provides an overview of the inclusion criteria through a flow diagram.

Identification

Initial results identified through ProQuest (n= 127,037)

Initial results identified through Scopus (n= 30,401).



Screening



Records unavailable (n = 14)

Records after duplications removed (n = 151)

Eligibility

Records excluded (n = 63)

Full text records screened (n = 137)

Included



**Fig. 1 Search inclusion criteria**
